# The *Lactobacillus* Bile Salt Hydrolase Repertoire Reveals Niche-Specific Adaptation

**DOI:** 10.1128/mSphere.00140-18

**Published:** 2018-05-30

**Authors:** Sarah O’Flaherty, Alexandra Briner Crawley, Casey M. Theriot, Rodolphe Barrangou

**Affiliations:** a Department of Food, Bioprocessing & Nutrition Sciences, North Carolina State University, Raleigh, North Carolina, USA; b Department of Population Health and Pathobiology, College of Veterinary Medicine, North Carolina State University, Raleigh, North Carolina, USA; University of Iowa

**Keywords:** *Lactobacillus*, bile acid, bile salt hydrolase, gastrointestinal tract, niche, penicillin V acylase, probiotic, therapeutic

## Abstract

Various *Lactobacillus* species have been reported to deconjugate bile acids in the gastrointestinal tract (GIT) through the action of bile salt hydrolase (BSH) proteins. This function contributes to altering the gut microbiota composition and bile metabolism and detoxification and to lowering cholesterol levels. Here, we investigated the *Lactobacillus* BSH repertoire across 170 sequenced species. We used hidden Markov models to distinguish between BSH and closely related penicillin-V acylase (PVA) proteins. Even though BSH and PVA proteins have very different target substrates, they share high sequence similarity and are often misannotated. We determined that 82/170 (48.24%) species encoded PVA proteins, 39/170 (22.94%) species encoded BSH proteins, and 8/170 (4.71%) species encoded both BSH and PVA proteins, while 57/170 (33.53%) species encoded neither. Mapping the occurrence of BSH-encoding species onto a phylogenetic tree revealed that BSH-encoding lactobacilli primarily adopt the vertebrate-adapted lifestyle but not the environmental or plant-associated subsets. Phylogenetic analysis of the BSH sequences revealed two distinct clades, several conserved motifs, and the presence of six previously reported active-site residues. These data will guide future mechanistic studies of BSH activity and contribute to the development and selection of BSH-encoding *Lactobacillus* strains with therapeutic potential.

**IMPORTANCE** Bile acids play an integral role in shaping the gut microbiota and host physiology by regulating metabolic signaling, weight gain, and serum cholesterol and liver triglyceride levels. Given these important roles of bile acids, we investigated the presence of bile salt hydrolase (BSH) in *Lactobacillus* genomes representing 170 different species, determined strain- and species-specific patterns of occurrences, and expanded on the diversity of the BSH repertoire in this genus. While our data showed that 28% of *Lactobacillus* species encode BSH proteins, these species are associated mainly with vertebrate-adapted niches, demonstrating selective pressure on lactobacilli to evolve to adapt to specific environments. These new data will allow targeted selection of specific strains of lactobacilli and BSH proteins for future mechanistic studies to explore their therapeutic potential for treating metabolic disorders.

## Abstract (precis)

Bile acids play an integral role in shaping the gut microbiota and host physiology by regulating metabolic signaling, weight gain, and serum cholesterol and liver triglyceride levels. Given these important roles of bile acids, we investigated the presence of bile salt hydrolase (BSH) in *Lactobacillus* genomes representing 170 different species, determined strain- and species-specific patterns of occurrences, and expanded on the diversity of the BSH repertoire in this genus. While our data showed that 28% of *Lactobacillus* species encode BSH proteins, these species are associated mainly with vertebrate-adapted niches, demonstrating selective pressure on lactobacilli to evolve to adapt to specific environments. These new data will allow targeted selection of specific strains of lactobacilli and BSH proteins for future mechanistic studies to explore their therapeutic potential for treating metabolic disorders.

## INTRODUCTION

Bile acids are synthesized by the liver from cholesterol and are essential for lipoprotein, glucose, drug, and energy metabolism (1, 2). Primary bile acids are made by the host and make their way through the small intestine, where 95% of bile acids are absorbed in the terminal ileum through the activity of the enterohepatic system (3). The remaining 5% that enter the large intestine can be modified by members of the gut microbiota to form secondary bile acids (4). Bile acids function to emulsify bile lipids such as cholesterol, facilitating adsorption into the bloodstream through the intestinal epithelium (3). In addition to facilitating adsorption of fats and cholesterol, bile acids can directly shape host physiology in a variety of ways, including acting as signaling molecules for the nuclear receptor farnesoid X receptor (FXR) (5, 6) and the G-protein-coupled TGR5 receptor (7). Given the dynamic role that bile acids play in regulating the host and gastrointestinal tract (GIT), bile acids and their derivatives represent promising targets for treating various metabolic disorders such as obesity, diabetes, and metabolic syndrome (3).

Primary and secondary bile acids can be conjugated with either glycine or taurine (8). Bile salt hydrolase (BSH) proteins then cleave the peptide linkage, releasing the amino acid group from the steroid core and the unconjugated bile acid (9). In particular, members of the gut microbiota such as lactobacilli have been studied for BSH activity because they encode BSH proteins (10–12). In fact, a common probiotic function or suggested characteristic of probiotic lactobacilli is often reported as their ability to deconjugate bile acids present in the GIT (13). It is thought that this function contributes to colonization and survival of bacteria in the GIT, in addition to providing positive attributes such as lowering cholesterol levels and impacting host signaling (9). While there has been much focus on lactobacilli that encode BSHs, their mechanism of action and precise contribution to GIT homeostasis remain elusive. Some *bsh* mutants have been generated that showcase growth defects in the presence of conjugated bile acids *in vitro*, suggesting that BSH proteins are important for bile tolerance in the GIT, although this is yet to be explored *in vivo* (11, 12). Through a metagenomic analysis of BSH activity in the human gut microbiome, Jones et al. (14) showed that gut microbes with BSH activity exhibited increased survival in the GIT and demonstrated enhanced resistance to conjugated bile acids. Additional work with gnotobiotic-raised mice implicated BSH in modified plasma bile acid profiles, modulation of pathways involved in lipid metabolism, and metabolic signaling (15). In the same study, conventionally raised mice with enhanced expression of BSHs displayed reduced weight gain and serum cholesterol and liver triglyceride levels and a reduced obesity phenotype (15). Given the importance of lactobacilli as members of the gut microbiota and as probiotic bacteria that have therapeutic potential in animals and humans for treatment of GIT-related disorders, we determined the BSH repertoire across 170 *Lactobacillus* species with publicly available genome sequences. This analysis builds on the recent determination of 213 *Lactobacillus* genomes by Sun et al. (16) and establishes a basis for understanding the role of gut bacteria in impacting bile acid composition in the host.

Despite recent advances in genome sequencing technologies and the continuing development of increasingly powerful bioinformatics tools, some of the bacterial genomic "dark matter" remains elusive, and functional annotation of bacterial genomes is incomplete and occasionally incorrect. Actually, annotating *bsh* sequences is complicated given the high levels of sequence homology with the closely related penicillin-V acylase (PVA) enzymes, which hydrolyze penicillin-V. These sequence similarities reflect overlap in the chemical bonds for which they show affinity and enzymatic activity. Indeed, both proteins are members of the choloylglycine hydrolase family, which comprises linear amide C-N hydrolases (Pfam PF02275). Therefore, *bsh* and *pva* genes are often confused for one another and misannotated. Previous work has taken this into account to improve methods for annotation of BSH and PVA proteins using hidden Markov models (HMMs) for Gram-positive bacteria (17) and a binding site similarity-based system for both Gram-positive and Gram-negative bacteria as well as archaea (18). Here, we also used HMMs for our analysis and focused on a detailed overview of the BSH repertoire in lactobacilli across 170 different species. We determined conserved domains within the proteins and revealed niche-specific adaptation for both PVA and BSH proteins by correlating their phylogenetic distribution with their lifestyle niche. These results open new avenues for the characterization of BSH activity, provide a basis for future mechanistic studies of the role of bile in the GIT, and guide the selection and development of probiotic *Lactobacillus* species with therapeutic potential.

## RESULTS

### Identification of BSH and PVA sequences in lactobacilli using BLASTX and HMM searches.

Knowledge of the BSH and PVA content of lactobacilli is valuable in the arena of probiotic, microbiome, and host-GIT interactions. Given the therapeutic potential of BSH proteins and of the *Lactobacillus* strains that encode them, we determined the BSH repertoire of lactobacilli through an *in silico* analysis of the genome content of 170 *Lactobacillus* species available in the NCBI database (see Table S1 in the supplemental material). We used a reference set of 26 previously described BSH sequences (Table 1) to search against a database of complete and draft *Lactobacillus* genomic sequences at NCBI. The resulting data were filtered to remove open reading frames encoding proteins less than 100 amino acids (aa) in length (as BSH proteins are approximately 320 aa in length) and hits with less than 30% amino acid sequence identity to the members of our reference group (see Fig. S1 in the supplemental material). Hidden Markov models (HMMs) were then constructed based on the BSH reference set and a PVA reference set (composed of eight sequences; see Table 1) to differentiate likely BSHs from likely PVAs in our data set. E values were used to determine whether our identified proteins more closely matched BSH proteins or PVA proteins (Fig. S1; see also Table S2). This analysis resulted in identification of 490 putative BSH proteins and 1,149 predicted PVA proteins. In addition, a phylogenetic tree of the BSH and PVA proteins showed a distinctive dichotomy between PVA and BSH proteins, which validates the selected reference sets and our approach (Fig. S2). We determined that across 170 *Lactobacillus* species, 82 species (48.24%) encoded PVA proteins, 39 species (22.94%) encoded BSH proteins, 8 species (4.71%) encoded both BSH and PVA proteins, and 57 (33.53%) species encoded neither.

10.1128/mSphere.00140-18.1 FIG S1  Flow chart of work flow used to identify the BSH and PVA content of lactobacilli. Download FIG S1, PDF file, 0.2 MB. Copyright © 2018 O’Flaherty et al. 2018 O’Flaherty et al. This content is distributed under the terms of the Creative Commons Attribution 4.0 International license.

10.1128/mSphere.00140-18.2 FIG S2  Phylogenetic tree of likely PVA and BSH proteins. Clusters from CD-HIT 80-cluster analysis of the data set showed two distinct clades for the likely BSH and PVA proteins. The likely BSH clade is shown in blue, and the likely PVA clade is shown in black. Download FIG S2, PDF file, 0.4 MB. Copyright © 2018 O’Flaherty et al. 2018 O’Flaherty et al. This content is distributed under the terms of the Creative Commons Attribution 4.0 International license.

10.1128/mSphere.00140-18.4 TABLE S1  Details of the 170 *Lactobacillus* species, representative strains, and metadata for the cladogram. Download TABLE S1, XLSX file, 0.02 MB. Copyright © 2018 O’Flaherty et al. 2018 O’Flaherty et al. This content is distributed under the terms of the Creative Commons Attribution 4.0 International license.

10.1128/mSphere.00140-18.5 TABLE S2  HMM search model results of likely BSH and likely PVA proteins. Download TABLE S2, XLSX file, 0.1 MB. Copyright © 2018 O’Flaherty et al. 2018 O’Flaherty et al. This content is distributed under the terms of the Creative Commons Attribution 4.0 International license.

**TABLE 1:** BSH and PVA reference sets

Species	Strain	Size (no. of aa)	Accession no.
BSH reference set			
Lactobacillus acidophilus	NCFM	325	YP_193782
Lactobacillus acidophilus	NCFM	325	AAV42923.1
Lactobacillus animalis	Unknown	324	WP_010690294.1
Lactobacillus buchneri	NRRL B-30929	327	AEB72500
Lactobacillus fermentum	NCDO 394	325	AEZ06356
Lactobacillus gasseri	ATCC 33323	316	ABJ59469.1
Lactobacillus gasseri	ATCC 33323	325	ABJ60345.1
Lactobacillus johnsonii	PF01	316	ABQ01980
Lactobacillus johnsonii	100–100	326	AF297873_1
Lactobacillus johnsonii	100–100	316	AAC34381
Lactobacillus johnsonii	NCC533	326	AAS09178
Lactobacillus johnsonii	NCC533	325	AAS08969
Lactobacillus johnsonii	NCC533	316	AAS08038
Lactobacillus plantarum	MBUL69	324	ACG76118
Lactobacillus plantarum	WCFS1	324	CCC80500
Lactobacillus plantarum	LP80	325	S51638
Lactobacillus plantarum	ST-III	324	ADO00098
Lactobacillus plantarum	ST-III	338	ADN97280
Lactobacillus plantarum	ST-III	328	ADN99975
Lactobacillus plantarum	ST-III	317	ADN99333
Lactobacillus reuteri	100–23	325	EDX41535
Lactobacillus salivarius	UCC118	316	YP_536688
Lactobacillus salivarius	NRRL B-30514	324	JX120368
Lactobacillus salivarius	JCM1046	325	ACL98204
Lactobacillus vaginalis	Unknown	325	WP_056974571
Lactobacillus vaginalis	Unknown	325	WP_040530699.1
			
PVA reference set			
Lactobacillus brevis	ATCC 367	325	Q03PK6
Lactobacillus brevis	ATCC 367	318	Q03NN7
Lactobacillus brevis	ATCC 367	327	Q03P51
Lactobacillus salivarius	UCC118	325	Q1WUK8
Lactobacillus sakei	23K	330	Q38Z70
Lactobacillus plantarum	WCFS1	338	Q890F5
Lactobacillus plantarum	WCFS1	328	Q88SP0
Lactobacillus plantarum	WCFS1	317	Q88UC9

### CD-HIT clustering of BSH proteins.

To delve deeper into the 490 potential BSH proteins identified in the analysis described above, we next concentrated on our BSH data set and clustered the BSH proteins to remove redundant entries and group highly similar sequences from our data set. We chose to cluster at 95% identity threshold, which resulted in 57 distinct clusters of BSH proteins, wherein proteins within a cluster shared at least 95% amino acid sequence identity. Manual analysis of the CD-HIT (cluster database at high identity with tolerance) data also facilitated identification of strains within *Lactobacillus* species that did not encode BSH proteins (Table 2; see also Table S3). We determined that only 39 of the 170 *Lactobacillus* species encoded BSH proteins; while the majority (64%) encoded a single BSH, some actually carried multiple BSH proteins (28% encoding one or two BSH proteins and 8% up to three BSH proteins; see Table 2). This analysis also revealed that the percentages of strains that encoded BSH proteins within a species ranged between 4.35% and 100%, reflecting variability not only between but also within species. Noteworthy, these percentages vary widely with the sequencing biases often observed in the NCBI database (Table 2), with content that varies over time as more genomic data are added.

10.1128/mSphere.00140-18.6 TABLE S3  Analysis of the CD-HIT 95% cluster results. Download TABLE S3, XLSX file, 0.02 MB. Copyright © 2018 O’Flaherty et al. 2018 O’Flaherty et al. This content is distributed under the terms of the Creative Commons Attribution 4.0 International license.

**TABLE 2:** Overview of the BSH content in lactobacilli^a^

Species	Total no. of strains	No. of strains with BSH	% of strains with BSH	No. of strains with:		
				1 BSH	2 BSH	3 BSH
Lactobacillus acidophilus	16	15	93.75	0	15	0
Lactobacillus agilis	2	2	100.00	1	1	0
Lactobacillus amylovorus	6	6	100.00	5	1	0
Lactobacillus animalis	3	3	100.00	3	0	0
Lactobacillus antri	1	1	100.00	0	1	0
Lactobacillus apodemi	1	1	100.00	0	1	0
Lactobacillus aviarius	15	15	100.00	14	1	0
Lactobacillus coleohominis	2	2	100.00	2	0	0
Lactobacillus crispatus	26	24	92.31	23	1	0
Lactobacillus delbrueckii	30	4	13.33	4	0	0
*Lactobacillus equicursoris*	2	2	100.00	2	0	0
Lactobacillus frumenti	1	1	100.00	1	0	0
Lactobacillus gallinarum	2	1	50.00	1	0	0
Lactobacillus gasseri	21	21	100.00	1	13	7
Lactobacillus gigeriorum	1	1	100.00	1	0	0
*Lactobacillus gorillae*	1	1	100.00	1	0	0
Lactobacillus hamsteri	1	1	100.00	1	0	0
Lactobacillus helveticus	23	1	4.35	1	0	0
Lactobacillus hominis	1	1	100.00	0	1	0
Lactobacillus ingluviei	3	1	33.33	1	0	0
Lactobacillus intestinalis	1	1	100.00	0	0	1
Lactobacillus johnsonii	9	9	100.00	1	5	3
Lactobacillus kalixensis	1	1	100.00	0	1	0
Lactobacillus kefiranofaciens	3	1	33.33	1	0	0
Lactobacillus kitasatonis	1	1	100.00	1	0	0
Lactobacillus mucosae	8	8	100.00	8	0	0
Lactobacillus murinus	3	3	100.00	3	0	0
Lactobacillus oris	4	3	75.00	3	0	0
Lactobacillus panis	1	1	100.00	1	0	0
Lactobacillus plantarum	182	147	80.77	147	0	0
Lactobacillus reuteri	30	28	93.33	28	0	0
Lactobacillus rogosae	1	1	100.00	0	1	0
Lactobacillus ruminis	14	12	85.71	12	0	0
Lactobacillus saerimneri	2	1	50.00	1	0	0
Lactobacillus salivarius	14	11	78.57	8	3	0
Lactobacillus secaliphilus	1	1	100.00	1	0	0
Lactobacillus taiwanensis	1	1	100.00	1	0	0
Lactobacillus ultunensis	1	1	100.00	1	0	0
Lactobacillus vaginalis	1	1	100.00	1	0	0

a Data are based on analysis of the CD-Hit clusters at 95% identity.

### **Mapping the occurrence of BSH and PVA proteins onto a** *Lactobacillus*** phylogenetic tree.**

**FIG 1:**
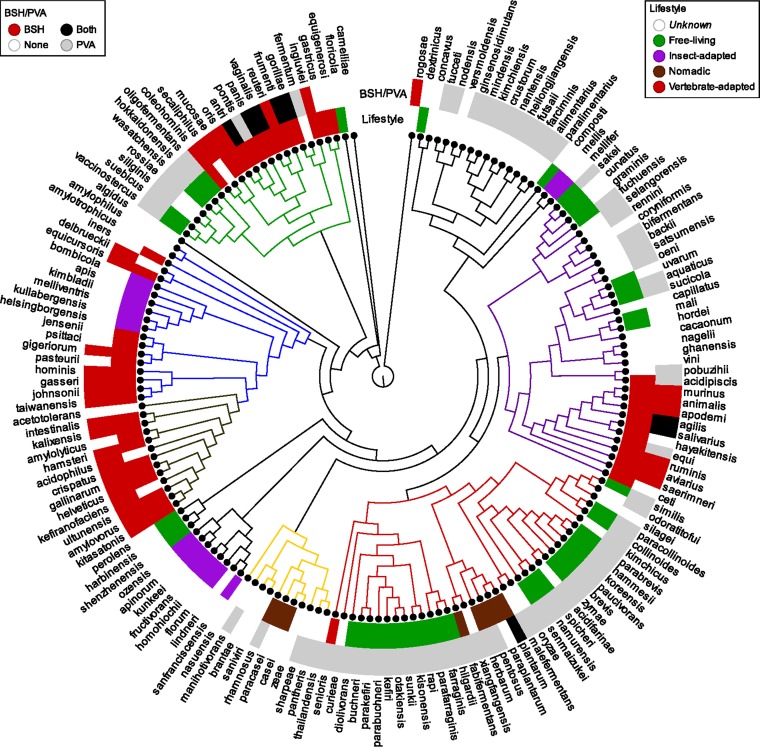
Phylogenetic tree of 170 *Lactobacillus* species. A phylogenetic tree was constructed for 170 representative *Lactobacillus* species based on the pyruvate kinase gene sequence for each genome. The colors of the groups follow those previously described (38): Lactobacillus animalis group is indicated in purple, Lactobacillus vaginalis group in green, Lactobacillus buchneri group in red, Lactobacillus rhamnosus group in yellow, Lactobacillus acidophilus group in maroon, and Lactobacillus gasseri group in blue. The inner metadata layer maps lifestyle designations as described by Duar et al. (20). The outer metadata layer maps the presence or absence of BSH and PVA proteins with respect to each of the 170 *Lactobacillus* species. Details of the reference strains used for each species and the metadata are located in Table S1.

A phylogenetic tree was created for the 170 species of lactobacilli on the basis of the pyruvate kinase enzyme sequence (Fig. 1) using the method recently described by Brandt and Barrangou (19, 38). A metadata layer of unknown, free-living, insect-adapted, nomadic, or vertebrate-adapted lifestyles was added as recently described by Duar et al. (20). The presence and absence of BSH and PVA proteins was mapped to the 170 *Lactobacillus* species (Fig. 1). The majority (84.62%) of species encoding BSH proteins mapped to the vertebrate-adapted lifestyle (with a minority mapping to unknown [12.82%] and nomadic [2.56%] lifestyles), whereas the PVA proteins mapped to free-living (40.24%), unknown (40.24%), vertebrate (13.41%), and nomadic (6.10%) lifestyles. Neither BSH-containing species nor PVA-containing species mapped to the insect-adapted lifestyle (Fig. 1). This distribution pattern likely reflects evolutionary pressure on vertebrate-associated species to preferentially encode BSH proteins.

### Alignment, phylogenetic tree, and identification of conserved motifs in *Lactobacillus* BSH protein sequences.

**FIG 2:**
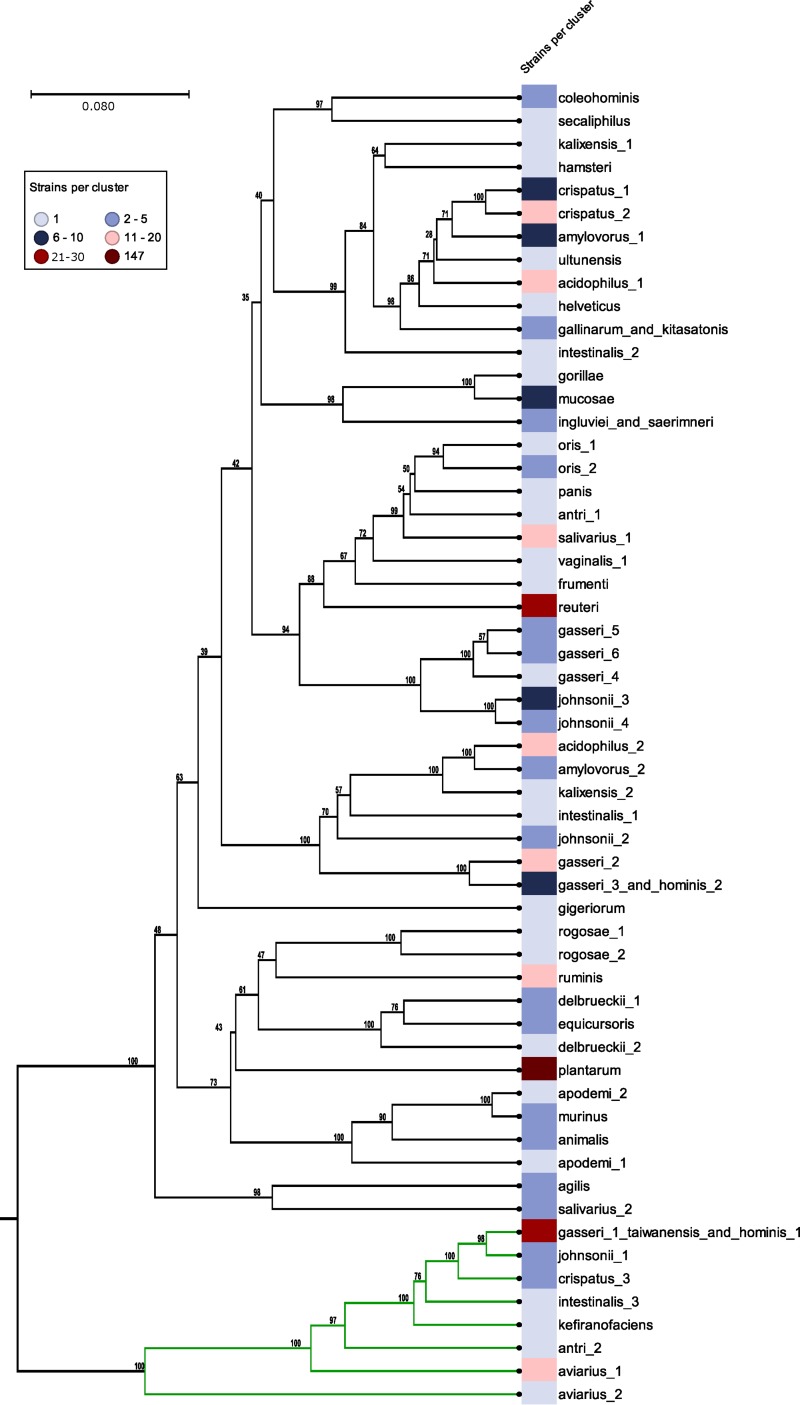
BSH phylogenetic tree for lactobacilli. A representative BSH protein from each of the 57 CD-HIT clusters was used in an alignment that was subsequently used to construct a phylogenetic tree. Each cluster is named in accordance with the BSH-containing species as indicated. Details of these clusters and strains can be found in Table S3. The number of strains per cluster is indicated in the metadata (Table 2). The bootstrap values are shown at the nodes. The two major clades are shown in black and green.

An alignment performed with one representative BSH protein sequence from each of the 57 clusters identified in the CD-HIT analysis was subsequently used to construct a phylogenetic tree of the BSH proteins. Two major clades were identified, with 49 clusters in the first clade and 8 clusters in the second (Fig. 2). The cluster groups were named for the selected species constituting each group. In addition, we mapped the number of strains per cluster encoding a BSH in that representative cluster group (Fig. 2). In the case of Lactobacillus plantarum, each of the 147 strains was found to encode one BSH, signifying that all 147 BSH proteins were located within one cluster and shared at least 95% identity. Conversely, the probiotic species Lactobacillus acidophilus was shown to encode two distinct BSHs that cluster into separate groups (Fig. 2) that shared 57% sequence identity (Table S4). In contrast, the data corresponding to the BSH content of Lactobacillus gasseri, an autochthonous species of the GIT (21), were diverse and distributed across six distinct cluster groups (Fig. 2). Five of these six cluster groups were located within the major clade and shared between 88% and 94% identity (Table S4). The sixth cluster group (gasseri_1_taiwanensis_and_hominis_1), which mapped to the smaller clade, shared only 33% to 38% sequence identity with the other groups (Table S4). However, the majority of L. gasseri strains encode two BSHs (Table 2). Taken together, these results indicate that BSH diversity is variable across species, lifestyles, and evolutionary paths.

10.1128/mSphere.00140-18.7 TABLE S4  Percent identity matrix of the 57 representative BSH proteins from the 57 clusters. Download TABLE S4, XLSX file, 0.02 MB. Copyright © 2018 O’Flaherty et al. 2018 O’Flaherty et al. This content is distributed under the terms of the Creative Commons Attribution 4.0 International license.

**FIG 3:**
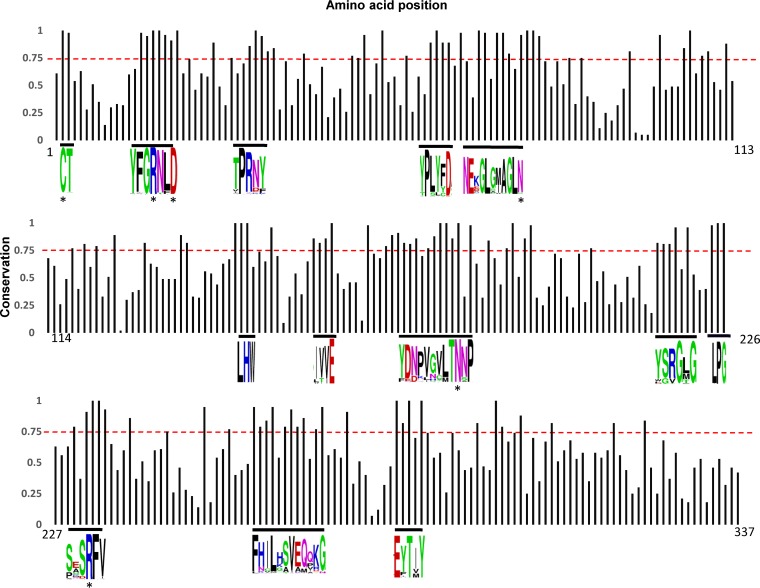
Conservation analysis of the BSH amino acid sequence. The alignment of the representative BSH proteins from the 57 clusters from the clustered data set was analyzed for conserved amino acid motifs. A conservation score of 0.75 or higher is indicated by a dashed red line. Motifs and conserved amino acids are indicated by the WebLogo. An asterisk indicates the previously described conserved active-site residues.

Next, the alignment of the 57 representative proteins from the CD-HIT cluster analysis was used to investigate and determine the occurrence of conserved motifs within BSH protein sequences (Fig. 3; see also Fig. S3). Motifs containing at least three amino acids, each with a conservation value of 0.75 or higher, are indicated (Fig. 3). A total of 12 motifs were identified, five of which contained previously reported conserved active-site residues (17, 22). The sixth active-site residue (corresponding to Cys2) was also determined to be conserved in our alignment (Fig. 3). As expected, the majority of the six active-site residues (indicated by asterisks) were located at the N-terminal region. Our data confirm and also expand upon previous results observed with smaller data sets (17).

10.1128/mSphere.00140-18.3 FIG S3  Alignment of the 57 representative BSH proteins from the BSH CD-HIT cluster analysis. Alignment of one representative protein sequence from each of the 57 clusters is shown. Cluster names are indicated on the left. Download FIG S3, PDF file, 0.6 MB. Copyright © 2018 O’Flaherty et al. 2018 O’Flaherty et al. This content is distributed under the terms of the Creative Commons Attribution 4.0 International license.

### Transcriptional profiles of *bsh* genes.

**FIG 4:**
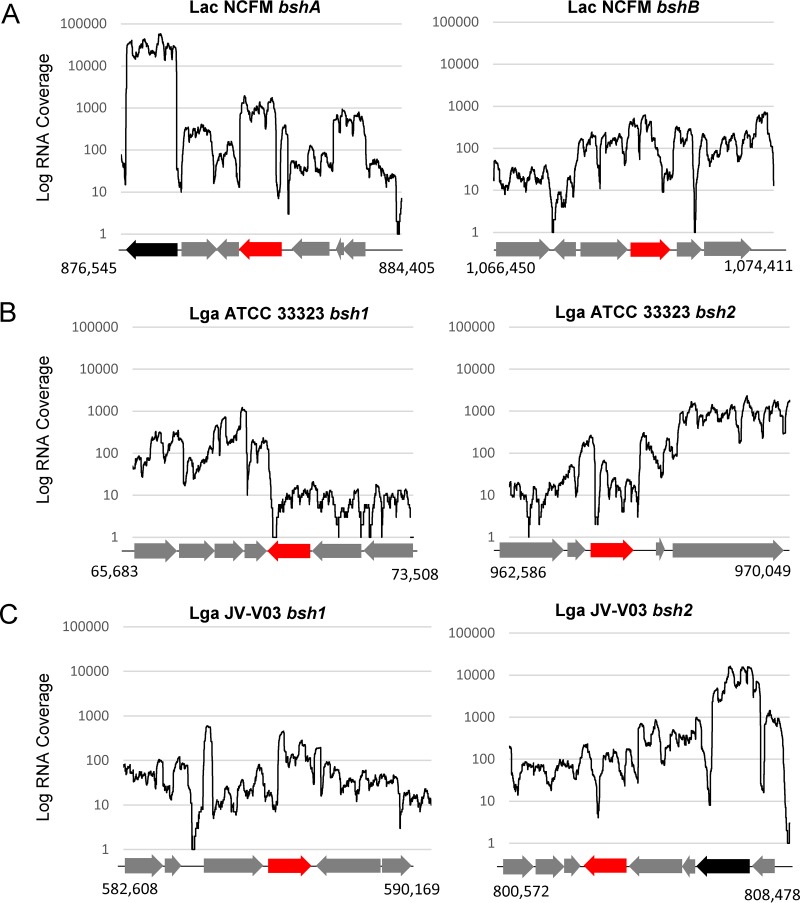
Transcriptional profiles of the *bsh* genes and surrounding genes for (A) L. acidophilus (Lac) NCFM *bshA* (LBA0892) and *bshB* (LBA1078), (B) L. gasseri (Lga) ATCC 33323 *bsh1* (LGAS_RS00260) and *bsh2* (LGAS_RS04710), and (C) L. gasseri JV-V03 *bsh1* (HMPREF0514_RS02865) and *bsh2* (HMPREF0514_RS03900). The red arrows indicate the *bsh* genes, the black arrows indicate the enolase genes, and the gray arrows indicate other genes. The genome coordinates for each segment of the genome are indicated for each region.

We used transcriptome sequencing (RNA-seq) data from three of the aforementioned distinct clusters, namely, L. acidophilus NCFM, L. gasseri ATCC 33323, and L. gasseri V-V03, to determine the genetic location and transcriptional profiles of various *bsh* genes (Fig. 4). We determined that the *bshA* gene was more highly expressed than the *bshB* gene in L. acidophilus NCFM and that the two genes were similarly expressed in the two L. gasseri strains (Fig. 4). There was no synteny in the context of the coding regions for the studied *bsh* genes (Fig. 4), although L. acidophilus NCFM *bshA* and L. gasseri JV-V03 *bsh2* were located two genes downstream of the enolase gene, which is one of the most highly expressed genes in both genomes (Fig. 4). While these transcriptional profiles do not necessarily reflect conditions within the GIT, these data show that all six *bsh* genes across the three strains were expressed in MRS broth and that these *bsh* genes are not part of operons but are monocistronic.

## DISCUSSION

Efforts to unravel the contribution of the gut microbiome to human health have rapidly expanded in the past decade, with the gut microbiome linked to many facets of well-being and disease, including response to medications (23), cancers (24), depression (25), and cardiovascular health (26). Empowered by the ready availability of genomics and transcriptomic and bioinformatics technologies, we can now study in depth the role of individual members of this consortium and investigate the correlations between the specific genotypes and phenotypes which can be ascribed to specific biochemical pathways and metabolites. Lactobacilli are members of the gut microbiome; while some species, such as L. gasseri, are considered autochthonous (21), others are transient or ingested as probiotic bacteria present in fermented foods and/or supplements due to their documented beneficial effects (13). Given the recent increase in the availability of whole-genome sequences of lactobacilli, we sought to mine all available lactobacilli with sequenced genomes in the public database for BSH proteins.

The focus of this study was to identity lactobacilli that encode BSH proteins, determine strain- and species-specific patterns of occurrences, and understand the diversity of the BSH repertoire in lactobacilli across 170 different species. Using a reference set of BSH and PVA proteins, we were able to distinguish between likely BSH proteins and likely PVA proteins using an HMM-based approach. Importantly, we did not rely on previous annotations but rather used a sequence-similarity-based approach to search available *Lactobacillus* genome sequences. The clear dichotomy and divergence between the resulting BSH proteins and PVA proteins was evident from the two distinct clades in the phylogenetic tree (Fig. S2). The cluster analysis was then deployed to condense redundant sequences and group highly similar sequences, resulting in a set of 57 distinct clusters. The majority (53 of 57) of clusters were species specific, though four clusters were composed of BSH proteins from two to three species. Analysis of the 57 clusters revealed two distinct clades of BSH proteins. Additionally, the results of the cluster analysis of the BSH proteins in lactobacilli suggest that BSH activity was acquired through horizontal gene transfer and support previous observations (10). Conserved motifs, encompassing six previously documented active sites, were identified. The most N-terminally located active-site residue (corresponding to Cys2) has been shown through site-directed mutagenesis to be essential for BSH activity (27). The identification of these motifs provides a basis for future functional studies designed to reveal their contribution to hydrolase activity. Biochemical characterization of the clusters should determine whether there are differences between the clusters with respect to hydrolase activities. It should also be confirmed that all of the clusters maintained specificity for bile acids and were not penicillin-hydrolyzing proteins. For three strains, including one L. acidophilus strain and two L. gasseri strains, we showed by RNA-seq that both *bsh* genes were transcribed during the exponential-growth phase in laboratory medium. This suggests that these genes are not solely homologs but likely would show differences with respect to activity and/or affinity *in vivo*. Further analyses are needed to determine whether these BSH proteins have redundant functions or have synergistic effects in hydrolyzing bile acids.

Our analysis determined that BSH proteins are far from ubiquitous in lactobacilli and are in fact encoded by a minority of species compared to PVA proteins. Once we determined the presence or absence of BSH in *Lactobacillus* species through *in silico* analysis, we investigated the correlation between the BSH-encoding pattern and the species lifestyle across the *Lactobacillus* phylogenetic tree, using a recently described method (19). It is noteworthy that this approach is efficient for comparing and contrasting genotypes (i.e., *bsh* and *pva* presence/absence) with phenotypes (i.e., lifestyle). Superimposing our occurrence data on the phylogenetic tree of 170 *Lactobacillus* species, we noted a clear correlation between the strains encoding *bsh* and their presumed lifestyle niche, with *bsh-*encoding lactobacilli correlating with a vertebrate-adapted lifestyle. These results are consistent with previous studies of BSH activity in the human gut microbiome reflecting evolutionary selective pressure for GIT-associated bacteria (14). In this case, we show that this specifically applies to diverse *Lactobacillus* species that encode BSH proteins and occur in the GIT of vertebrates. Interestingly, our data also showed that the *Lactobacillus* species that mapped to the insect-adapted lifestyle encoded neither BSHs nor PVAs, indicating a lack of selective pressure for these species to require choloylglycine hydrolase activity in this niche.

Within the 28% of *Lactobacillus* species encoding BSH proteins, the majority (64%) encoded one BSH protein, while 28% encoded one or two proteins. Our data confirm previous results by Lambert et al. (17); for example, in the case of L. plantarum WCSF1, four BSH proteins were originally annotated in the genome of L. plantarum WCSF1. *In silico* analysis predicted that L. plantarum would in fact encode only one BSH protein (17). Subsequent experimental work confirmed this observation, with those authors proposing the remaining three genes encode PVA proteins (11). In our study, we also predicted that L. plantarum would encode a single BSH protein. The L. plantarum BSH protein shared 58% identity at most with all other BSHs identified in this study; however, the active-site residues were conserved. In fact, L. plantarum was by far the most highly represented species in our data set, with 182 different strains. Among those, we determined that 147 encoded BSH proteins. These data indicate either that there is a lack of strain diversity within the available sequenced L. plantarum strains and/or that the L. plantarum species have evolved to encode highly conserved BSH proteins that shared at least 95% identity across all 147 strains even though L. plantarum mapped to the nomadic lifestyle.

In contrast, through our BSH analysis of lactobacilli, we also determined that both Lactobacillus helveticus and Lactobacillus delbrueckii contain a minority of strains that encode BSH proteins. L. delbrueckii is a species typically used in yogurt fermentation and is an example of a *Lactobacillus* species undergoing genome decay (28, 29), given its protocooperation with Streptococcus thermophilus in milk (29). In the case of L. delbrueckii, 4 strains among a total of 30 strains in the database were found to encode one BSH; 3 of those strains were located in the same cluster group, while the fourth shared 78% identity, indicating low sequence diversity between these proteins. Similarly, of the 23 L. helveticus strains included in our study, just one was shown to encode a BSH protein. Even though L. helveticus is mapped to the vertebrate-adapted lifestyle (20), this species has been shown to be evolving toward domestication in dairy environments because of its historical use in cheese fermentations (28, 30). Through genome decay, L. helveticus has lost many of the GIT-associated attributes associated with its close relative L. acidophilus, such as BSH activity, complex sugar uptake and catabolism capability, and presence of mucus-binding proteins (30). Our data reflect this evolutionary path for dairy-associated L. helveticus strains given that only one strain was determined to encode a single BSH.

While tolerance of bile acids is often mentioned as a required or desired probiotic attribute, relatively little has been done beyond determination of crude growth curves and plate assays to understand the multifaceted function of BSH proteins. This study has expanded our knowledge of the BSH content of lactobacilli downward to the strain level. In addition, we have described diversity and occurrence with respect to comparisons between strains and species. The identification of the BSH repertoire of lactobacilli will not only aid in future mechanistic studies of the BSH proteins themselves but will also contribute to the selection and study of strains and BSH proteins with therapeutic potential in the GIT. Understanding the metabolic activities of important gut bacteria, their impact on other bacteria, and their contributions to host health will guide probiotic strain selection and alteration of the gut microbiome toward a healthier state.

## MATERIALS AND METHODS

### BLASTX analysis to determine BSH and PVA sequences in lactobacilli.

The complete nucleotide repertoire for lactobacilli with whole-genome sequence data was downloaded from NCBI in April 2017, resulting in 1,096 entries. The data were built into a local BLAST Lactobacillus database. BSH (26 sequences) and PVA (8 sequences) reference sets were curated based on previously characterized BSH and PVA proteins (Table 1). The BSH reference set was used for a BLASTX search against the Lactobacillus database. BLASTX was used for searches to prevent biases in gene-calling algorithms and to detect potentially truncated sequences. Search results were filtered using custom code to return the correct coding sequence at each genetic locus with at least 30% identity to a reference protein. Only proteins that were at least 100 aa in length were used for further analyses.

### Hidden Markov model (HMM) search to differentiate between BSH and PVA candidates.

Prior to using the HMM, both the BSH and the PVA reference sets were aligned using MUSCLE (31) in biopython (http://biopython.org/) to make a Stockholm multisequence alignment (MSA) file. The Stockholm MSA file was then used to run the HMMER (32) search on the output of the BLASTX search to determine potential BSH and PVA proteins. All proteins were compared to the BSH and PVA HMMs. Custom code was used to compare the E values from the BSH and PVA to filter and to remove potential false positives (E values less than 1e−99) as well as to select which profile each protein more closely matched. This process was able to differentiate likely BSH proteins from likely PVA proteins, which was not possible using BLAST alone.

### CD-HIT clustering of BSH and PVA proteins.

CD-HIT clustering (33, 34) was used to condense similar and identical sequences prior to alignment and phylogenetic tree analysis. The newly identified BSH data set was used to create clusters of BSH proteins that shared at least 95% homology. Geneious workflows were then used to extract the sequences from the CD-HIT analysis for alignments, tree construction of BSH proteins, and determination of conserved amino acids (Geneious Biomatters version 10 [35] and CLC Genomics workbench 9.5.3 [Qiagen]).

### Construction of a phylogenetic tree for lactobacilli.

One representative genome was selected for each of the 170 species of lactobacilli identified in the Lactobacillus database downloaded from NCBI (see Table S1 in the supplemental material). Methods described by Brandt and Barrangou (19) were used to construct a phylogenetic tree for the 170 lactobacilli. Briefly, the nucleotide sequence for the pyruvate kinase enzyme was identified for each of the 170 genomes using the "annotate from genome" function and extracted from each representative genome using Geneious workflows (Geneious version 10 [35]). The 170 pyruvate kinase nucleotide sequences were then imported into CLC Genomics (CLC Genomics workbench 9.5.3 [Qiagen]). CLC Genomics was used to align the 170 sequences and construct the phylogenetic tree for the 170 lactobacilli. The metadata feature was used to map the presence and absence of BSH or PVA proteins determined in this study to the phylogenetic tree. Lifestyle attributes for the *Lactobacillus* species in the phylogenetic tree were adapted from Duar et al. (20).

### RNA isolation, sequencing, and analysis.

Total RNA was isolated from L. gasseri strains (ATCC 33323 and JV_V03) grown to mid-log phase (optical density at 600 nm [OD_600_], ~0.6) in MRS broth at 37°C. Methods for RNA isolation and RNA sequence analysis were as described previously (36). Cells were harvested by centrifugation (1,717 × *g* for 10 min at room temperature), and total RNA was extracted using TRI reagent (Life Technologies, Inc., Carlsbad, CA) and purified with an RNeasy minikit (Qiagen, Valencia, CA). The cell pellets were resuspended in 1 ml of TRI reagent, added to a screw-cap tube containing 0.1-mm-diameter glass beads (Bio-Spec), and subjected to bead beating for 5 min (5 times each at 1-min intervals with 1 min on ice after each interval). Subsequently, 200 µl of chloroform was added to each sample. Samples were then left on ice for 20 min and centrifuged at 16,873 × *g* for 20 min at 4°C. The aqueous layer was transferred to a fresh tube, and RNA was purified with an RNeasy minikit (Qiagen) using the cleanup procedure according to the manufacturer’s instructions. DNA was removed by incubating samples with Turbo DNase as described by the manufacturer (Ambion Inc., Austin, TX), purified using an RNeasy minikit (Qiagen), and checked for integrity by capillary electrophoresis on an Agilent Bioanalyzer (Agilent Technologies, Santa Clara, CA).

Library preparation and sequencing were performed at the High-Throughput Sequencing and Genotyping Unit of the Roy J. Carver Biotechnology Center, University of Illinois at Urbana—Champaign. A Ribo-Zero kit (Bacteria) (Illumina, San Diego, CA) was used to remove rRNA followed by library preparation performed with a TruSeq Stranded RNA Sample Prep kit (Illumina, CA). The libraries were pooled in equimolar concentrations, and each pool was quantitated by the use of quantitative PCR (qPCR) and sequenced on one lane for 161 cycles using an Illumina HiSeq 2500 Ultra-High-Throughput sequencing system (with a read length of 160 nucleotides) and a HiSeq SBS sequencing kit (version 4). Fastq files were generated and demultiplexed with bcl2fastq v2.17.1.14 conversion software (Illumina). Adapter sequences were removed, and raw sequences were assessed for quality using Fast QC version 0.11.4 (http://www.bioinformatics.babraham.ac.uk/projects/fastqc/). Subsequent processes were performed with Geneious (35). Raw reads were trimmed to remove bases with an error probability limit of 0.001 (Phred score of 30), and reads of <20 nucleotides were removed. Sequences were mapped to their reference genomes using the Geneious mapper (35). The data set for L. acidophilus NCFM was from a previously published study (37).

### Accession number(s).

The accession numbers for the data are as follows: SRR6329247 (L. acidophilus NCFM), SRR6329248 (L. gasseri ATCC 33323), and SRR6763418 (L. gasseri JV-V03). The *bsh* genes for L. acidophilus NCFM have previously been annotated as LBA0892 (*bshA*) and LBA1078 (*bshB*) (12). For both the L. gasseri ATCC 33323 (LGAS_RS00260 and LGAS_RS04710) and JV-V03 (HMPREF0514_RS02865 and HMPREF0514_RS03900) *bsh* genes, we used the designations *bsh1* and *bsh2*, respectively, to represent the two genes in the genome.
