# Supplementary figures and images for: The Lactobacillus Bile Salt Hydrolase Repertoire Reveals Niche-Specific Adaptation
Source: mSphere. 2018 May 30;3(3):e00140-18. doi: 10.1128/mSphere.00140-18 (PMC5976879; doi:10.1128/mSphere.00140-18)

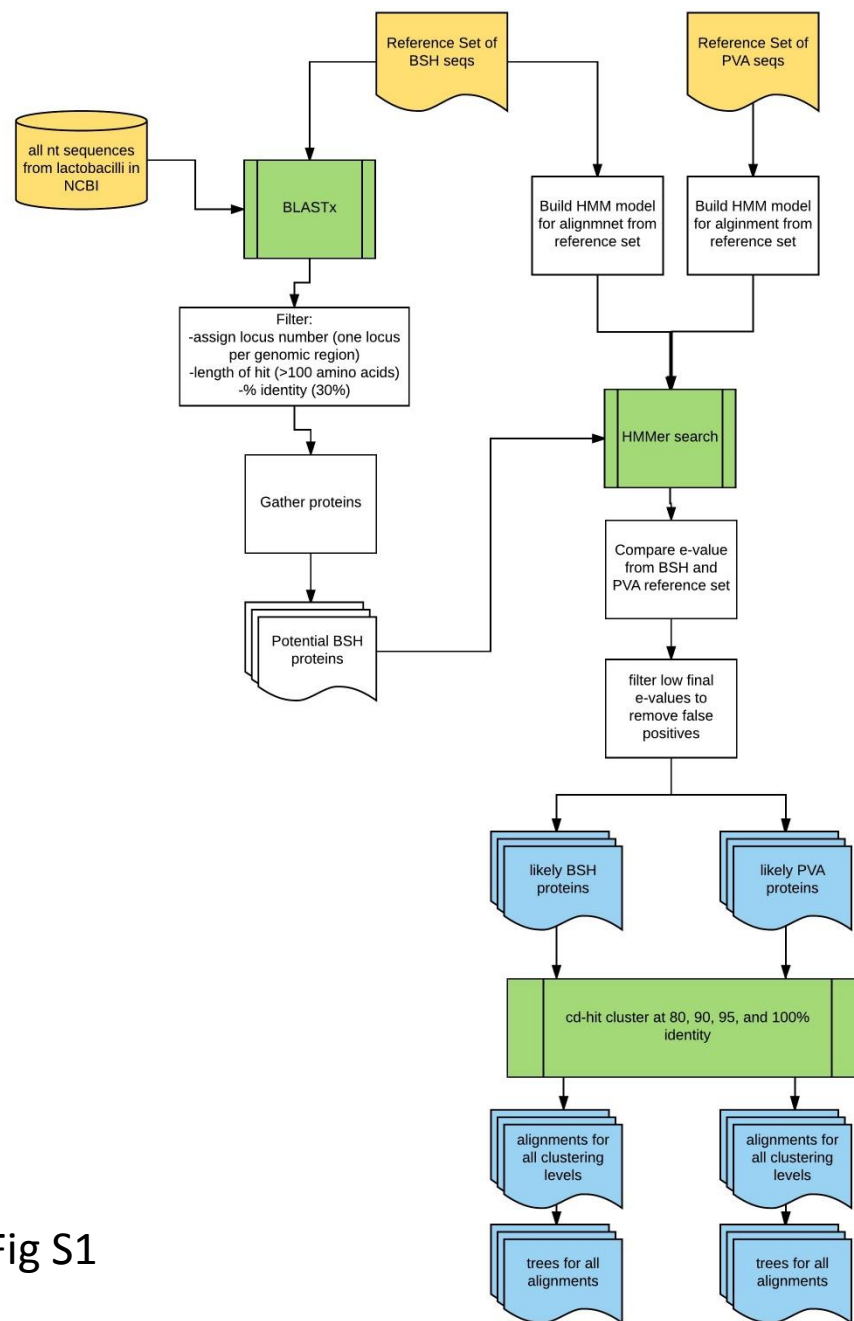

Fig S1

Supplement: FIG S1 [file sph003182556sf1.pdf]

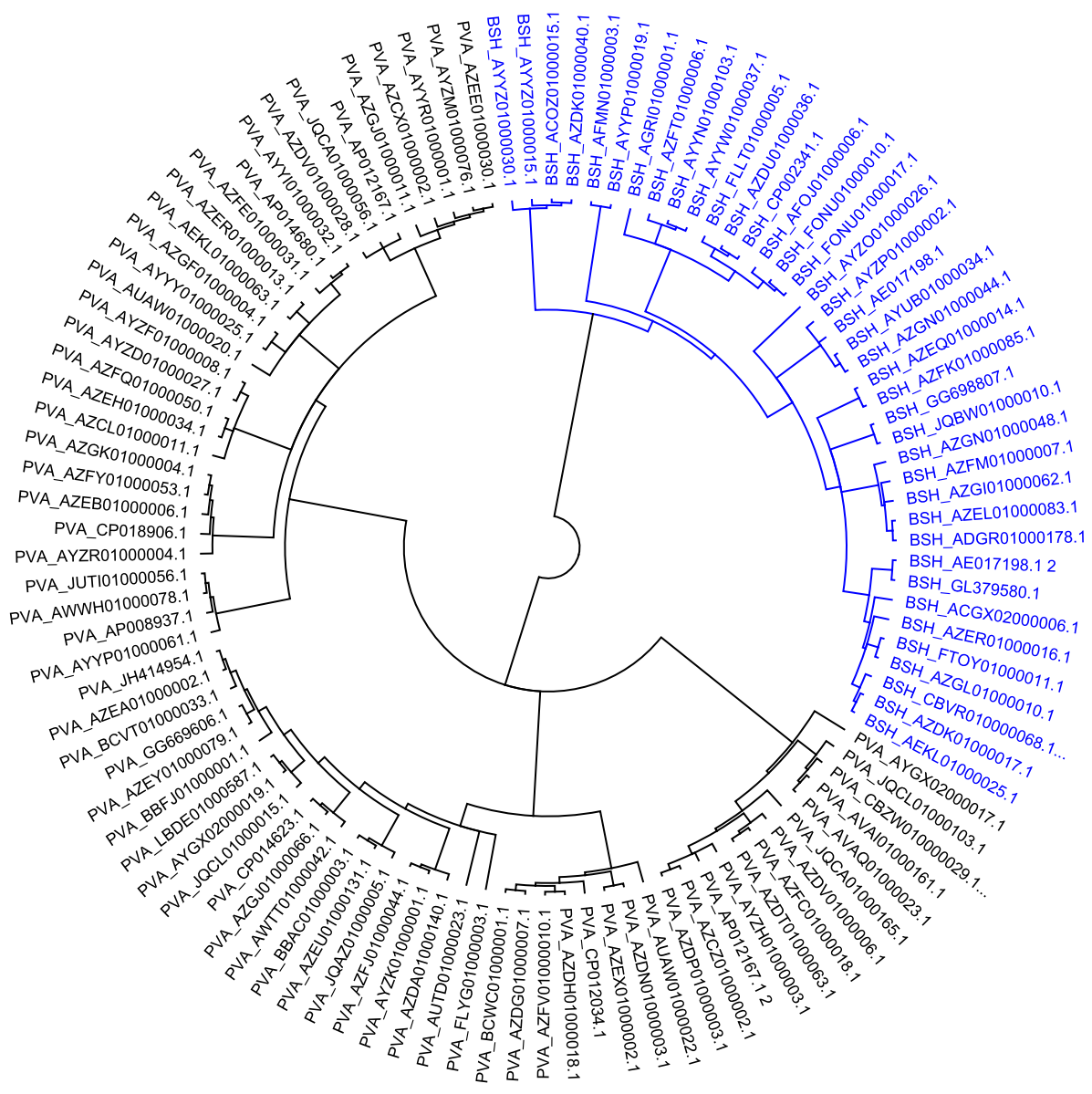

Supplement: FIG S2 [file sph003182556sf2.pdf]

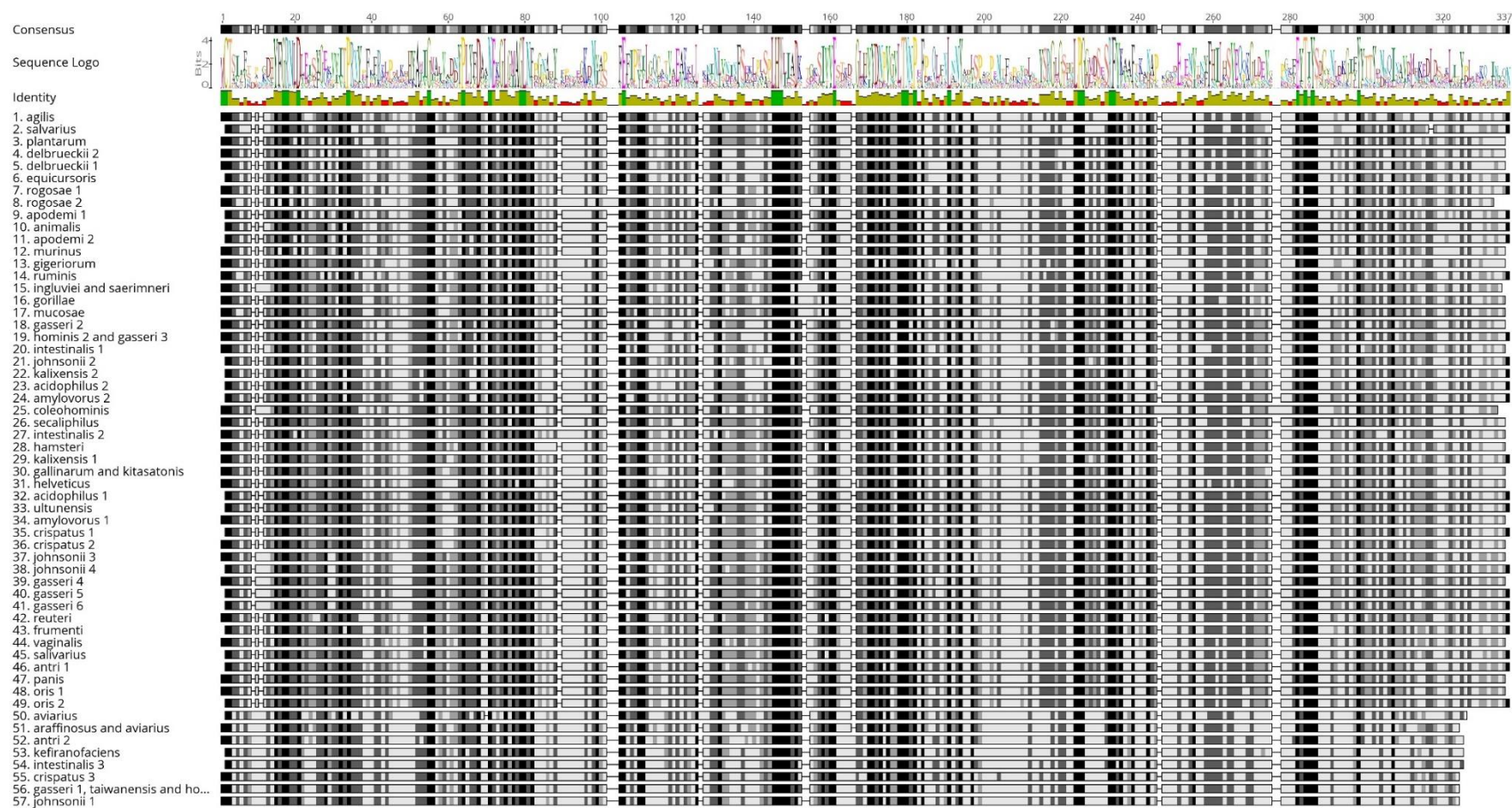

Fig S3

Supplement: FIG S3 [file sph003182556sf3.pdf]
